# Supplementary material for: Identification of tauopathy-associated lipid signatures in Alzheimer’s disease mouse brain using label-free chemical imaging
Source: Commun Biol. 2024 Oct 17;7:1341. doi: 10.1038/s42003-024-07034-3 (PMC11487145; doi:10.1038/s42003-024-07034-3)
Supplement: Supplementary file 2 — Supplementary Information [file 42003_2024_7034_MOESM2_ESM.pdf]

Supplementary Information

Identification of tauopathy-associated lipid signatures in Alzheimer's disease mouse brain using label-free chemical imaging

Hao Meng,[1] Alicia Elliott,[1] Jessica Mansfield,[1] Michelle Bailey,[1] Mark Frogley,[2] Gianfelice Cinque,[2] Julian Moger,[1] Nick Stone,[1] Francesco Tamagnini,[3,4] and Francesca Palombo*[1]

[1] Department of Physics and Astronomy, University of Exeter, Exeter, EX4 4QL, UK.

[2] Diamond Light Source, MIRIAM beamline B22, Harwell Science & Innovation Campus, Didcot, OX11 0DE, UK.

[3] School of Pharmacy, University of Reading, Reading, RG6 6UB, UK.

[4] Centro Studi Biomedici, Università degli Studi della Repubblica di San Marino, Salita alla Rocca, 44 – 47890 San Marino Città, RSM.

∗ Corresponding author: F.Palombo@exeter.ac.uk.

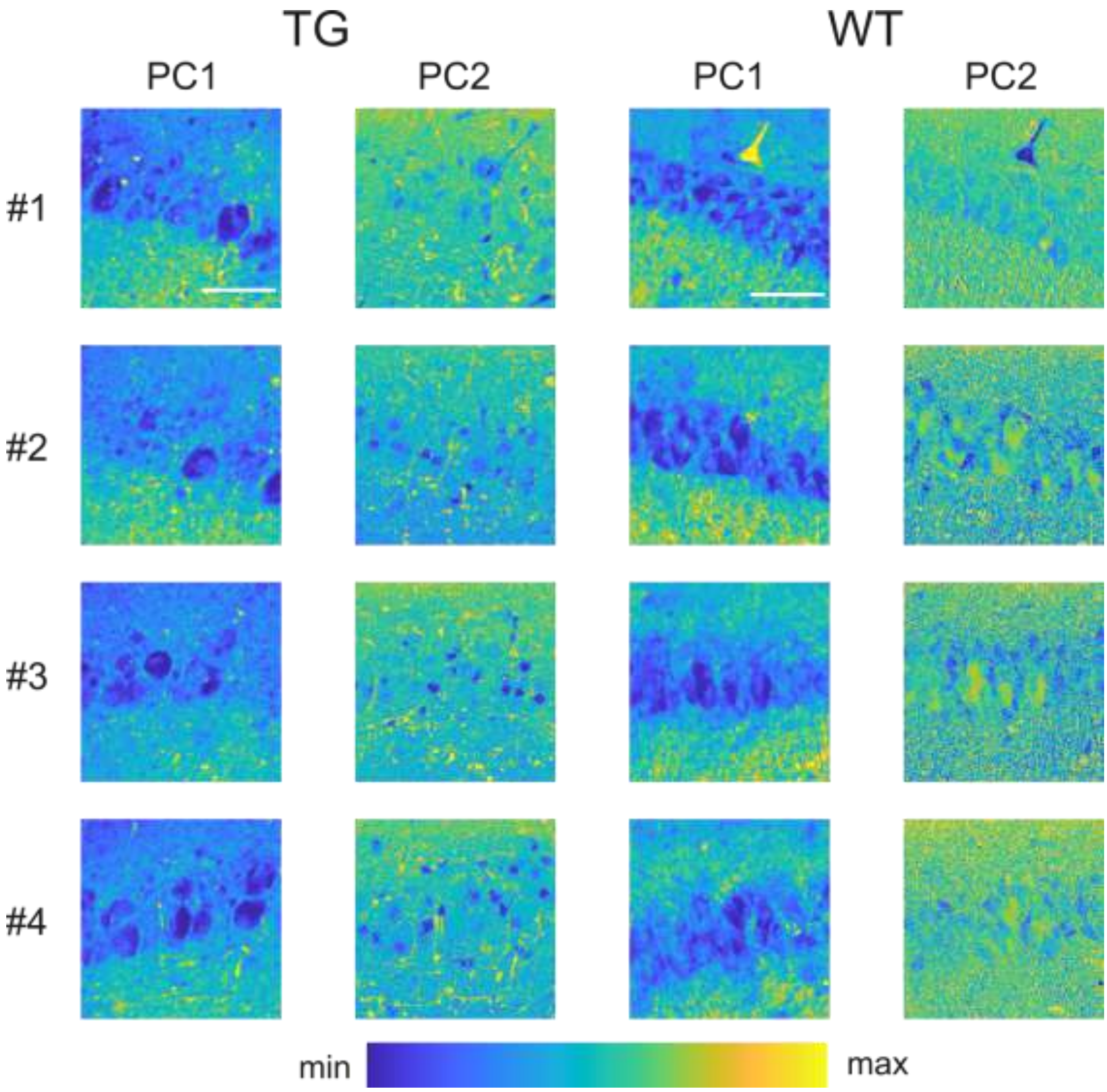

Figure SI-1 Results of SOM-PCA applied to SRS hyperspectral maps of TG and WT mouse brain hippocampal sections from four distinct ROIs. SOM-PCA scores of PC1 and PC2 of ROIs (#1 - #4) in TG and WT samples. Scale bar: 20µm; colour coding: blue (low) to yellow (high).

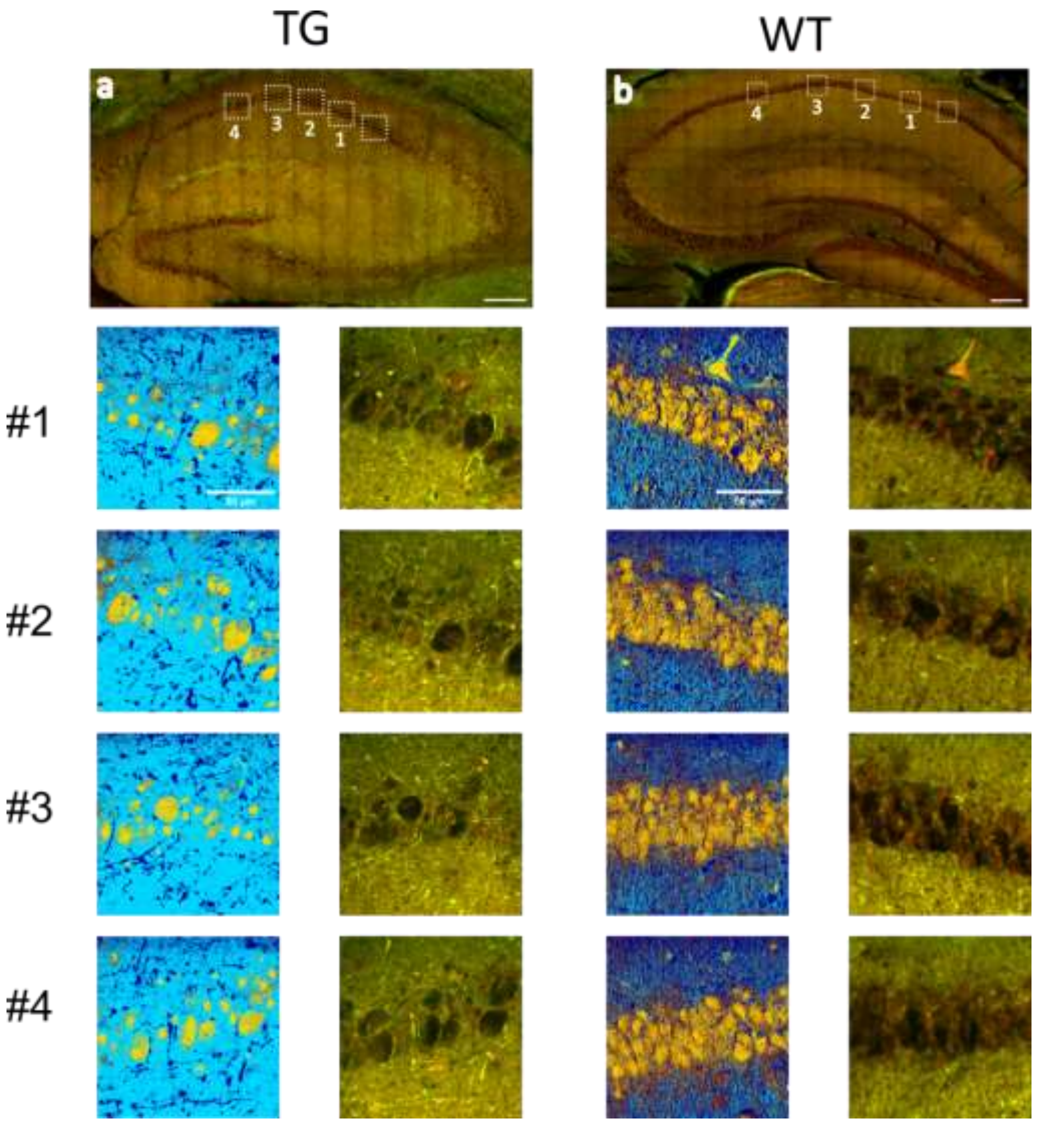

Figure SI-2 Results of common k-means cluster analysis of SRS hyperspectral maps of TG and WT mouse brain hippocampal sections across four distinct ROIs (a,b) Merged images of the whole TG and WT mouse brain hippocampus at the wavenumber of 2844 cm^{-1} and 2930 cm^{-1}. Scale bars: 200µm. Dashed boxes indicate five distinct ROIs where SRS maps were acquired. (Bottom panels: #1 - #4) Images derived from common k-means analysis with 4 clusters for each ROI in TG and WT. Corresponding merged images are listed for comparison. Scale bars: 50µm.

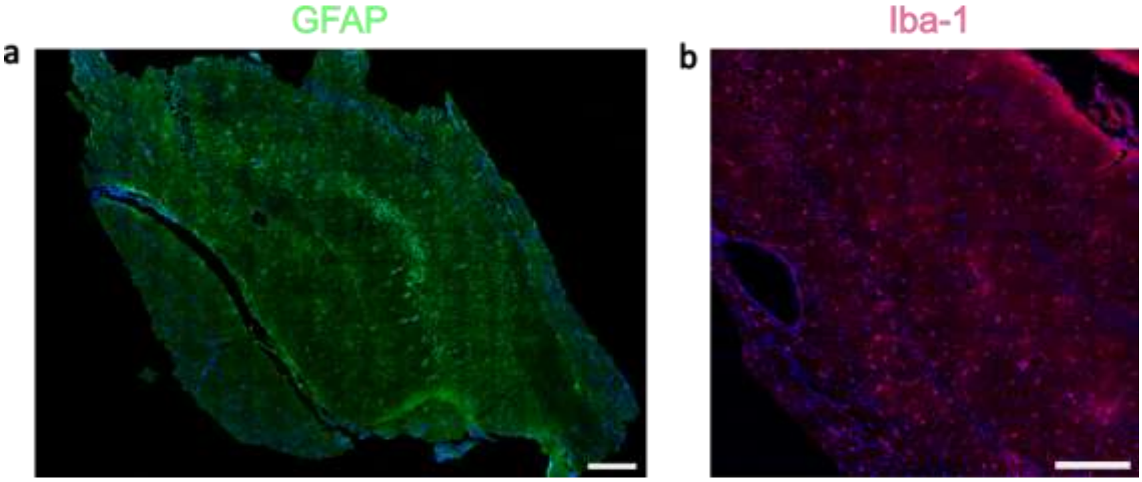

Figure SI-3 Fluorescence images of stained TG mouse hippocampal tissue sections. (a) GFAP (green) and DAPI (blue) co-staining. (b) Iba-1 (magenta) and DAPI (blue) co-staining. Scale bars: 200µm.

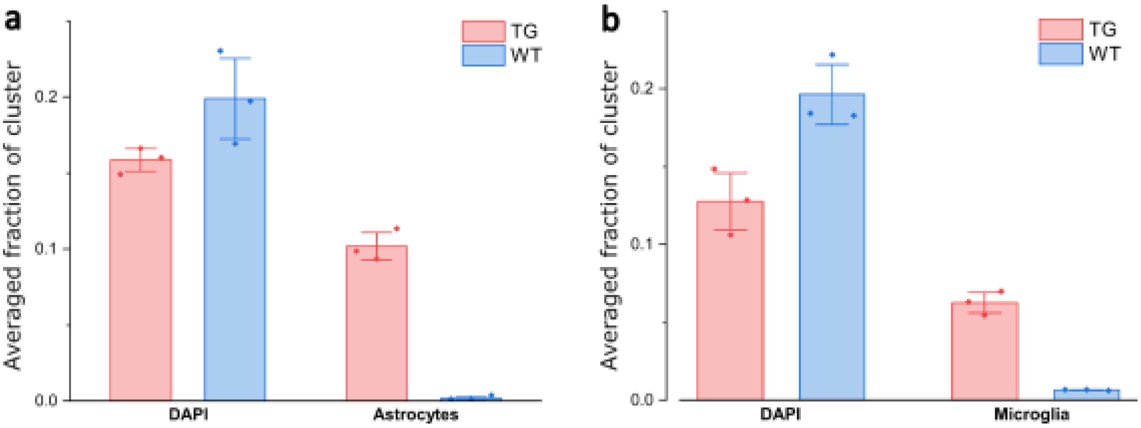

Figure SI-4 Fraction of staining for nuclei, activated astrocytes and microglia for five ROIs in TG and WT samples.

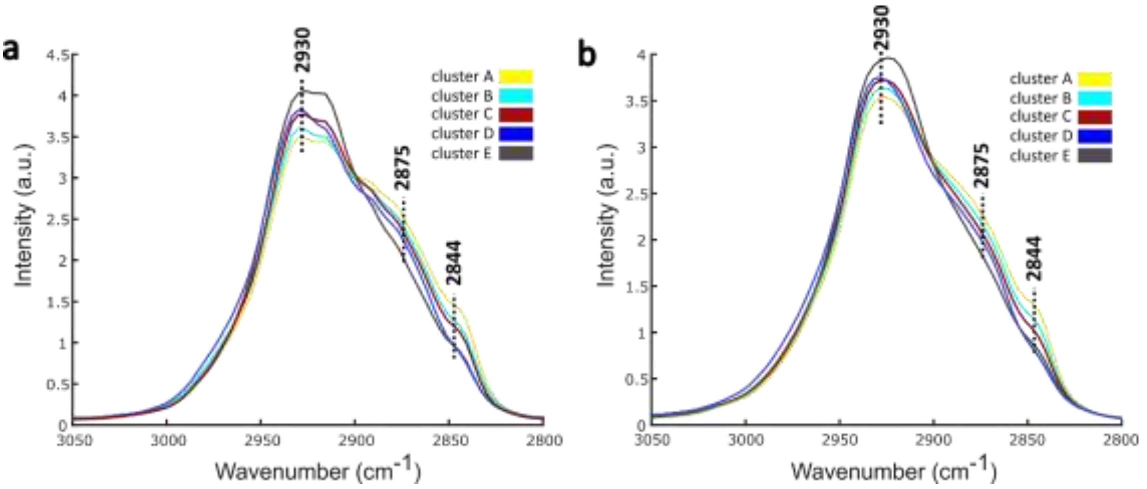

Figure SI-5 Cluster centroid spectra derived from k-means cluster analysis of TG samples stained with (a) GFAP and (b) Iba-1. Main peaks of *CH* stretching modes are labelled.

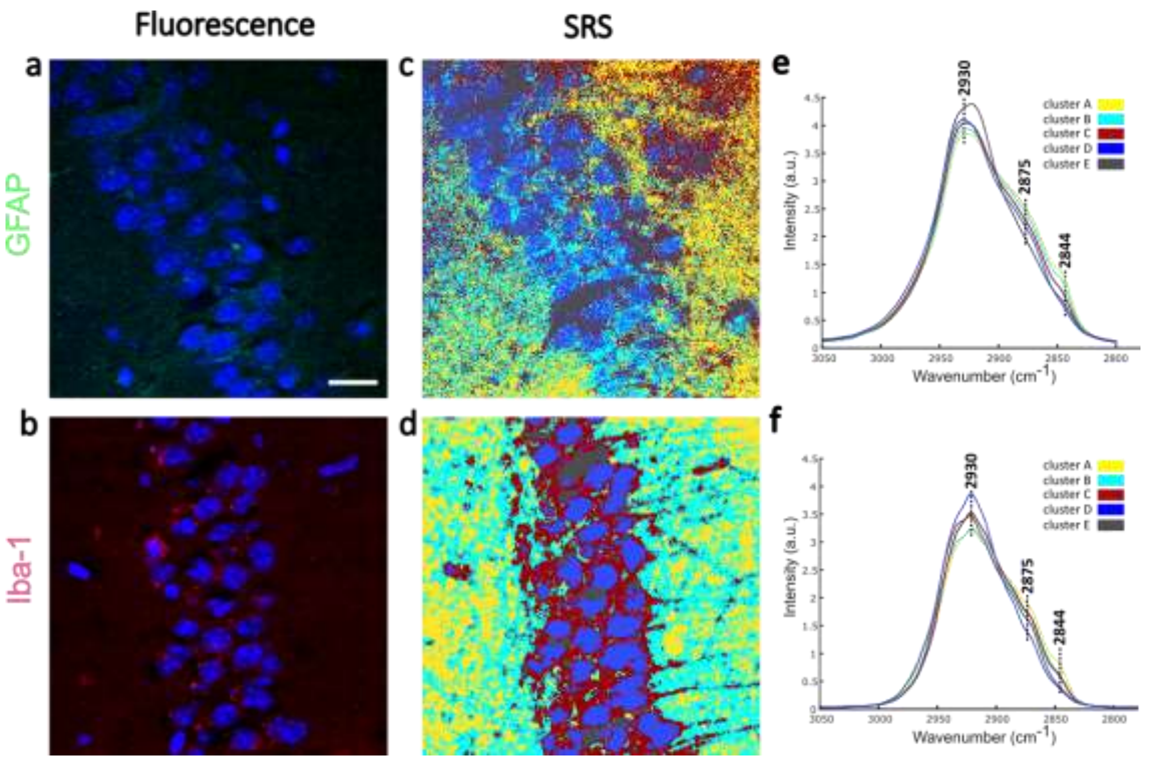

Figure SI-6 Images derived from correlative (a,b) immunofluorescence and (c,d) SRS microscopy on two WT tissue sections co-stained with DAPI (nuclei; blue) and either GFAP (astrocytes; green) or Iba-1 (microglia; magenta). (c,d) Spatial segmentation obtained from k-means cluster analysis with 5 clusters. Scale bar: 20µm. (e,f) Cluster centroid spectra derived from k-means cluster analysis. Main peaks of *CH* stretching modes are labelled.
